# In vitro shear bond strength over zirconia and titanium alloy and degree of conversion of extraoral compared to intraoral self-adhesive resin cements

**DOI:** 10.1038/s41405-023-00178-0

**Published:** 2023-12-09

**Authors:** Vincent Fouquet, Claire-Adeline Dantagnan, Sarah Abdel-Gawad, Elisabeth Dursun, Jean-Pierre Attal, Philippe François

**Affiliations:** 1 Université Paris Cité, Faculté de Santé, UFR d’Odontologie, Paris, France; 2 Innovative Dental Materials and Interfaces Research Unit (URP 4462), Montrouge, France; 3 Hôpital Louis-Mourier, AP-HP, Colombes, France; 4 Hôpital Pitié-Salpétrière, AP-HP, Paris, France; 5 Hôpital Henri-Mondor, AP-HP, Créteil, France; 6 Hôpital Charles-Foix, AP-HP, Ivry-sur-Seine, France; 7 Hôpital Bretonneau, AP-HP, Paris, France

**Keywords:** Dental materials, Prosthetic dentistry

## Abstract

**Objective:**

Evaluation of the Shear bond strength over zirconia and titanium alloy and degree of conversion of extraoral compared to intraoral self-adhesive resin cements.

**Materials and methods:**

Nine bonding protocols were carried out on zirconia 4Y-TZP and titanium alloy (Ti-6Al-4V). Seven resin cement (one extraoral and six intraoral) were tested in the shear bond strength test and the degree of conversion measurements.

**Results:**

The significantly highest value was obtained for Monobond Plus + Multilink Hybrid Abutment, the extraoral resin cement for both titanium alloy (35.1 MPa) and zirconia (32.9 MPa). For each resin, significantly higher DC values were obtained for the dual-cure mode compared with the self-cure mode. Regardless of the cure mode, Nexus Universal reached the highest DC (78.4%).

**Discussion/Conclusions:**

In this study, the extraoral self-curing resin cement showed the higher bond strength values on zirconia and titanium alloy when associated with a universal primer. Some intraoral dual-cure resin cements showed closed performances when used with universal primers. There is no direct correlation between the degree of conversion of the resin cement and the shear bond strength obtained on the prosthetic materials tested.

## Introduction

Zirconia and titanium alloys are widely used materials in prosthetic dentistry. In practice, they need to be carefully grit-blasted to achieve good micromechanical retention (50 µm aluminum oxides) [1]. Moreover, the use of functional phosphorylated monomers (10-Methacryloyloxydecyl dihydrogen phosphate (10-MDP), 10-methacryloyloxydecyl dihydrogen thiophosphate (MDTP), glycerol phosphate dimethacrylate (GPDM) and others) in resin cement enhances chemical adhesion [2–4], whereas the application of silane has no effect, given the absence of silica in zirconia and titanium alloy [5]. Among the bonding strategies currently available, there are three bonding methods used after grit-blasting. The first is the use of a dedicated prosthetic primer containing functional phosphorylated monomers and silane, called universal primer, then applying a resin cement [6–8]. The second method consists of replacing the specific prosthetic primer with a universal adhesive (that also contains functional monomers) and then applying a resin cement [9]. The third is to directly use a resin cement containing functional monomers as part of its formulation [10].

Zirconia can be bonded on dental tissues, such as cementing zirconia crowns on prepared teeth abutments (intraoral bonding) or on titanium base abutment (Ti-base) in prosthetic labs (extraoral bonding) [11]. Some resin cements are dedicated for intraoral use, whereas others are indicated for extraoral use. To follow the trend of simplifying procedures [12, 13], it would be relevant to determine whether intraoral dedicated resin cements could also be used as efficiently as than extraoral ones.

Depending on their chemical composition, these resin cements can be self-cured (chemopolymerized), light-cured (photopolymerized) or dual-cured (association of chemopolymerization and photopolymerization) [14]. There are clinical or laboratory situations in dentistry that do not allow efficient light curing (assembly of inlay cores, thick zirconia crowns, Ti-bases) and under which [15–17], resin polymerization relies almost only on the self-curing mode [18]. To our knowledge, there is only one study (in another context) that compares the bond strength of intraoral and extraoral resin cements to zirconia or titanium alloy in the current trend toward simplification [19], and few prior research investigates the role played by light curing in the curing process [20].

The aim of this study was first to evaluate the shear bond strength of one extraoral and various intraoral resin cements to zirconia and titanium alloy according to nine bonding protocols in self-curing mode (in order to assess their bonding strength in the least favorable polymerization mode) and then to measure their degree of conversion in self-curing mode or with additional light curing. The first null hypothesis was that there are no differences in shear bond strengths between the nine protocols tested resin for each substrate. The second null hypothesis was that there were no differences in the degree of conversion between the various resin cements in self-curing mode or with an additional light-cured mode.

## Materials and Methods

### Materials used

The materials used in this study are summarized in Table 1.

**Table 1:** Materials, abbreviations, manufacturers, batch number and chemical composition.

Types of materials	Names	Abbre-viation	Manufacturer	Batch Number	Composition
Extraoral resin cements	Multilink Hybrid Abutment	MHA	Ivoclar AG, Schaan, Liechtenstein	Z04K3Z	Monomer matrix: dimethacrylate, HEMA, camphorquinone. Inorganic fillers (aprox 36%): barium glass, ytterbium trifluoride, spheroid mixed oxide, titanium oxide.
Intraoral resin cements	G-Cem One	GC-O	GC Corporation, Tokyo, Japan	2207211	Fluoroalunimosiliciate glass, dimethacrylate, initiator, stabilizer, pigment, silicon dioxide, MDP SiO_2_, trimethoxysilane, 2-hydroxy-1,3-dimethacrylatoxypropane, 6-tert-butyl-2,4-xylenol, 2,6-di-tert-butyl-p-cresol, EDTA disodium salt dehydrate, vanadyl acetylacetonate, TPO, ascorbic acid, camphorquinone, MgO
	SpeedCem Plus	SCP	Ivoclar AG, Schaan, Liechtenstein	Z02H2V	UDMA, TEGDMA, PEGDMA, phosphoric acid ester, dibenzoyl peroxide, Ytterbium trifluoride, Barium glass, Silicon dioxide (0.1–7 μm average particle size)
	RelyX Universal	R-U	3 M Dental, St Paul, MN, USA	9214626	DMAs, Phosphorylated DMAs, novel amphiphilic redox initiator system, radiopaque fillers
	Panavia SA Universal	PSA-U	Kuraray-Noritake, Niigata, Japan	3N0013	Monomer (10-MDP, Bis-GMA, TEGDMA, HEMA), initiator. Methacrylate monomer, filler (silanated barium glass filler, aluminum oxide, silanated sodium fluoride), accelerator, pigment, silane coupling agent
	Nexus Universal	N-U	Kerr, Orange, CA, USA	8570950	Bisphenol A Diglycidyl Methacrylate, Proprietary Methacrylate, Triethylene Glycol Dimethacrylate, Urethane Dimethacyrlate, Ethoxylated Bisphenol A Dimethacrylate, Poly(oxy-1,2-ethanediyl), alpha,-hydro- omega-[(1-oxo-2-propen-1-yl)oxy]- ether with 2-ethyl-2-(hydroxymethyl)-1,3- propanediol
	Totalcem	TTC	Itena Clinical, Paris, France	4284-01HQBSEA2	UDMA, Bis-GMA, TEGDMA, 4-methacryloxyethyltrimellitic acid, barium glass, fumed silica
Universal primers	G-Multi Primer	GMP	GC Corporation, Tokyo, Japan	2104121	Ethanol, γ-MPTMS, 10-MDP, MDTP, Bis-GMA, TEGDMA
	Monobond Plus	MP	Ivoclar AG, Schaan, Liechtenstein	Z02S82	Ethanol, 3-trimethoxysilylpropylmethacrylate, 10-MDP (MDP), sulfide methacrylate
Universal adhesives	Scotchbond Universal Plus	SBUP	3 M Dental, St Paul, MN, USA	7910510	Bis-GMA, 10-MDP, 2-HEMA, Vitrebond copolymer, ethanol, water, initiators, fillers
	Clearfil Universal Bond	CUB	Kuraray-Noritake, Niigata, Japan	3P0296	Bis-GMA, HEMA, MDP, camphorquinone, colloidal silica, silane coupling agent, ethanol, water, hydrophilic aliphatic dimethacrylate
	Optibond Universal	OBU	Kerr, Orange, CA, USA	8305743	Acetone, 2-hydroxyethyl methacrylate, ethanol, glyceryl dimethacrylate
Zirconia	Katana STML	Z	Kuraray-Noritake, Niigata, Japan	DZUTL	4Y-TZP zirconia
Titanium alloy	Ti-6Al-4V	T	NeoNickel, Chassieu, France	N4512	Titanium alloy grade V (Ti6Al4V)

### Shear bond strength (SBS) test and failure mode

Nine bonding protocols (Table 2) were carried out on zirconia 4Y-TZP (Katana Zirconia STML, Kuraray-Noritake) and titanium alloy (Ti-6Al-4V) (NeoNickel, Chassieu, France) for a total of 18 variable groups. Ten samples were produced per variable groups.

**Table 2:** Bonding protocols tested.

Groups	Abbreviations	Bonding protocol
Monobond Plus + Multilink Hybrid Abutment	MP + MHA	A thin layer of MP was applied with a micro-tip applicator and dried with an air syringe. Then, MHA was applied from the syringe by an automix tip.
G-CEM One	GC-O	The GC-O was applied from the syringe by an automix tip.
G-Multi Primer + G-CEM One	GMP + GC-O	A thin layer of GMP was applied with a micro-tip applicator, dried with an air syringe. Then, GC-O was applied from the syringe by an automix tip.
Monobond Plus + SpeedCem Plus	MP + SCP	A thin layer of MP was applied with a micro-tip applicator and dried with an air syringe. Then, SCP was applied from the syringe by an automix tip.
Scotchbond Universal Plus + RelyX Universal	SBUP + R-U	A thin layer of SBUP was applied with a micro-tip applicator and dried with an air syringe. Then, R-U was applied from the syringe by an automix tip.
Panavia SA Cement Universal	PSA-U	The PSA-U was applied from the syringe by an automix tip.
Clearfil Universal Bond + Panavia SA Cement Universal	CUB + PSA-U	A thin layer of CUB was applied with a micro-tip applicator and dried with an air syringe. Then, PSA-U was applied from the syringe by an automix tip.
Optibond Universal + Nexus Universal	OBU + N-U	A thin layer of OBU was applied with a micro-tip applicator and dried with an air syringe. Then, N-U was applied from the syringe by an automix tip.
Totalcem	TTC	The TTC was applied from the syringe by an automix tip.

Blocks of zirconia and titanium alloy were included in self-curing acrylic resin and then abraded with water-cooled sandpaper (800 grits) to expose a flat surface (>7 mm2). All these samples were grit-blasted with Al_2_O_3_ (50 µm) for 10 s at 2 bars, rinsed with water/air spray, then with a 99% ethanol solution and dried.

On each sample (*n* = 180), a cylindrical Teflon mold was placed to build a 3 mm-high cylinder of resin cement with a flat base of 7 mm² (diameter = 3 mm). The tested bonding protocols were performed following the manufacturer’s instructions (which can lead to various protocols for a specific resin cement). The resin cement was set in self-curing mode (in the dark) under 50 g pressure for 60 min. Then, the mold was removed, and the excess, if present, was gently removed with a scalpel. All the samples were stored in demineralized water at 37 °C for 24 h. Detailed bonding protocols are summarized in Table 2.

For each group (*n* = 10), SBS values were determined in a universal testing machine (LRX, Lloyd Instruments, Fareham, UK). The shear force was applied at the resin cement/prosthetic material interface, with a chisel-shaped blade parallel to the prosthetic material surface. A cross-head speed of 0.5 mm/min was chosen.

The debonded specimens were observed under a binocular microscope (BZH10 Olympus, Hamburg, Germany) at 30× magnification, and the failure modes were classified according to the following four types: cohesive failure within the prosthetic material, adhesive failure at the interface between the resin cement and the prosthetic material, mixed failure (adhesive and cohesive failure within the prosthetic material), cohesive failure within the resin cement.

### Degree of conversion measurements

For each resin cement tested (*n* = 7), 6 cylindrical specimens (diameter 6 mm x height 3 mm) were made with Teflon molds: 3 were performed in with light-activation for 60 s at 1200 mW per square cm (Valo Grande lamp, Ultradent Products Inc, South Jordan, UT, USA) immediately after mixing, and 3 were performed only in self-curing mode (mixed in the dark). All samples were then stored in the dark in distilled water at 37 °C for one week before the degree of conversion (DC) measurement.

DC was determined using Fourier transformed infrared spectroscopy (FTIR) with a NicoletTM iS10 spectrometer (Thermo Fisher Scientific, Waltham, MA, USA) in attenuated total reflectance (ATR) mode following proposed protocol from previous publications [21]. These spectra were recorded with OMNIC software (Thermo Electron Corporation, Waltham, MA, USA). After measuring the background, all measurements were obtained in the spectral region of 500 to 4000 cm^−1^ under the following conditions: a resolution of 4 cm^−1^ and 32 internal scans per reading. For all resin cements analyzed, spectra in the unpolymerized state were also collected to serve as a reference. The spectra of each specimen were recorded 3 times.

For each spectrum, the height of the absorption band of the aliphatic C=C bond (1638 cm^−1^) and that of the aromatic C=C bond (1608 cm^−1^) were measured, first in the monomer state and then after the polymerization process. DC was determined by evaluating the change in the height ratio of the aliphatic C=C peak and the aromatic C=C peak during the polymerization process [22], according to the following formula:

$${DC}\,\left( \% \right)=\left(1-\frac{{Abs}\,\frac{C=C1638\, {{{{{\rm{cm}}}}}}^{-1}\,\left(P\right)}{C=C1608\, {{{{{\rm{cm}}}}}}^{-1}\left(P\right)}}{{Abs}\,\frac{C=C1638\, {{{{{\rm{cm}}}}}}^{-1}\,\left({NP}\right)}{C=C1608\, {{{{{\rm{cm}}}}}}^{-1}\,\left({NP}\right)}}\,\right)\, \times 100$$

*Abs: Absorbance, P: Polymerized, NP: Nonpolymerized*

### Statistical analysis

Normal distribution of SBS and DC values was confirmed by the Shapiro‒Wilk test, and the equality of variances was assessed using the Levene test before the other tests were performed. SBS and DC data were expressed as the mean values and standard deviations.

For SBS results, two one-way ANOVAs followed by Tukey’s post hoc test for each bonding substrate (zirconia or Ti-6Al-4V) were used to investigate the difference in SBS between the different groups. The failure mode was analyzed by Fisher’s exact test for single comparisons between groups and pairwise analysis.

For DC results, a 2-way ANOVA followed by Tukey’s post hoc test was performed for the factors “resin” and “mode of polymerization”. In all tests, the significance level was *p* < 0.05. Statistical calculations were performed using R software (R version 3.6.1, R Foundation for statistical computing, Vienna, Austria).

## Results

### SBS values and failure mode analysis

The mean SBS values and standard deviations are reported in Table 3.

**Table 3:** SBS values and standard deviation for each group tested.

Prosthetic materials	Bonding protocols	SBS in MPa (±SD)
Zirconia	MP + MHA	32.9 (±1.9)^**A**^
	GMP + GC-O	29.4 (±3.2)^**B**^
	GC-O	29.1 (±2.8)^**B**^
	SBUP + R-U	28.9 (±2.8)^**B**^
	MP + SCP	27.6 (±1.9)^**B,C**^
	PSA-U	24.7 (±1.6)^**C,D**^
	CUB + PSA-U	24.3 (±2.3)^**D**^
	OBU + N-U	24.3 (±2.1)^**D**^
	TTC	12.7 (±1.4)^**E**^
Ti-6Al-4V	MP + MHA	35.1 (±1.6)^**a**^
	GMP + GC-O	32.0 (±2.7)^**b**^
	MP + SCP	30.3 (±2.0)^**b,c**^
	PSA-U	29.2 (±2.7)^**b,c,d**^
	CUB + PSA-U	29.0 (±2.6)^**c,d**^
	SBUP + R-U	28.3 (±1.8)^**c,d,e**^
	OBU + N-U	26.7 (±1.7)^**d,e**^
	GC-O	25.9 (±1.8)^**e**^
	TTC	15.9 (±1.4)^**f**^

Values with the same letter in exponent are not significantly different at *p* < 0.05.

The significantly highest SBS value was obtained for MP + MHA, the extraoral resin cement for both Ti-6Al-4V (35.1 MPa) and zirconia (32.9 MPa). In contrast, TTC had the lowest SBS values regardless of the prosthetic material (15.9 MPa for Ti-6Al-4V and 12.7 MPa for zirconia).

The use of universal primer or universal adhesive did not significantly improve the bond strength on zirconia (29.1 MPa for GC-O *vs*. 29.4 MPa for GMP + GC-O; 24.7 MPa for PSA-U *vs*. 24.3 MPa for CUB + PSA-U), whereas it was significantly increased on titanium alloy for GC-O (25.9 MPa for GC-O *vs*. 32.0 MPa for GMP + GC).

Adhesive failures occurred in every group. No significant differences in the failure occurrences were shown between groups.

### Degree of conversion

The degree of conversion values obtained for each resin cement are reported in Table 4.

**Table 4:** Degree of conversion and standard deviation for each resin cement with self-cure and dual-cure protocols.

Polymerization mode	Resin cements	DC in% (±SD)
Dual-cure	R-U	73.3 (±0.0)^**B**^
	GC-O	75.2 (±0.0)^**B**^
	TTC	70.2 (±0.0)^**C**^
	N-U	78.4 (±0.0)^**A**^
	PSA-U	73.6 (±0.0)^**B**^
	SCP	73.1 (±0.0)^**B**^
	MHA	74.2 (±0.1)^**B**^
Self-Cure	R-U	68.4 (±0,0)^**C**^
	GC-O	64.3 (±0.1)^**D**^
	TTC	60.3 (±0.1)^**E**^
	N-U	69.1 (±0.1)^**C**^
	PSA-U	62.3 (±0.0)^**E**^
	SCP	68.4 (±0.0)^**C**^
	MHA	65.8 (±0.1)^**D**^

Values with the same letter in exponent are not significantly different at *p* < 0.05.

For each resin, significantly higher DC values were obtained for the dual-cure mode compared with the self-cure mode. Regardless of the cure mode, N-U reached the highest DC (78.4%), whereas TTC had the lowest DC (60.3%).

## Discussion

### Shear bond strength values and failure mode analysis

It is difficult to establish an immediate in vitro threshold bond strength, despite that such a measurement could ensure long-term clinical adhesion. However, long-term clinical studies show good bonding performance on zirconia and Ti-6Al-4V when the standard protocols are followed correctly [23–26].

In this study, macro-shear bond strength test was used to assess SBS values of the various adhesive protocols to Zirconia and Ti-6Al-4V. The macro-shear bond strength test is one of the two most frequently used tests, along with the micro-tensile bond strength test, to assess the strength of an adhesive procedure [27–29]. Some papers have compared the relevance of micro-tensile and macro-shear bond strength tests, showing that the resultant forces at the interface are neither pure shear for the macro-shear test [28], nor pure traction for the micro-traction test [28]. Although the micro-traction test is considered more representative than the macro-shear test, the latter offers a number of technical advantages in terms of sample preparation in the laboratory and was selected for this study.

Bonding strength values on Ti-6Al-4V and zirconia were measured concomitantly, due to the dual indication of these two materials in certain prosthetic indications such as bonding crowns on abutments in implantology. It is therefore interesting to know the performance of the same protocol on these two substrates. However, in mouth, the two materials have different clinical indications. Zirconia can be bonded in a large number of different fixed prosthetic indications [30, 31], while titanium is less frequently used in bonded indications, except in certain cases of intra-radicular posts or periodontal splints [32, 33].

The extraoral resin cement bonding protocol MP + MHA was chosen for study here because of its widespread use in the literature with good clinical retention results [34–36]. As a result, it is considered a gold standard when a prosthetic assembly is performed outside the mouth. In our study, the MP + MHA extraoral protocol led to the highest bond strength values over both zirconia and Ti-6Al-4V. The first null hypothesis is therefore rejected.

The two highest values found for each prosthetic material were MHA and GC-O using their dedicated universal primers (MP and GMP, respectively). There is a general trend of specific primers such as MP or GMP to be more effective than universal adhesives used as primers on titanium alloy and zirconia [37]. MP is a diluted ethanolic solution that consists of three mutually stable bonding monomers, silane, phosphoric acid agent and disulfide [38]. GMP has a slightly different composition with the presence of γ-MPTMS, 10-MDP, MDTP, bis-GMA and TEGDMA [39]. As zirconia and titanium alloys possess a high affinity to phosphoric acid in forming poorly soluble phosphates [40], these specific primers containing methacrylate monomers with a functional phosphoric-acid group result in a strong bond and hydrolysis resistance with them. Although the detailed adhesion mechanism remains largely unexplored, the bond between metal oxides and acid monomers is produced by intermolecular forces such as hydrogen bonds [41]. The most common acid monomer used to achieve this chemical bonding is 10-MDP, which is thus present in the two universal primers tested. This monomer is considered one of the most effective materials (if not the most effective) for creating chemical bonds with the two substrates studied [10, 42–44]. It is often combined with other molecules in universal primers for synergistic action and to obtain higher bond strength values on zirconia and nonprecious metals but also to make them effective on precious metals (sulfur compounds) or vitreous ceramics (presence of silane) [42]. These various mixtures within universal primers are suspected of being detrimental to achieving the higher interaction with our two substrates tested but are nevertheless commonly accepted as methods at least as reliable for bonding these substrates as tribochemical grit-blasting which consists in applying a thin layer of silica to the surface of the prosthetic element followed by the use of a primer containing silane [45, 46]. Other zirconia bonding processes have also been proposed in the literature and are currently being evaluated [47].

Here, all dental universal adhesives tested as an alternative to universal primers also contained 10-MDP or glyceryl dimetacrylates as functional monomers but led to lower bond strength values than when a universal primer was used. All curing procedures were carried out in a dark room with a self-curing mode to avoid activating light-curing polymerization of resin cements. Under these conditions, universal adhesives used as nonspecific primers were not light-cured either. This could also contribute to the lower bond strength values, even though manufacturers report self-cure contact with their associated resin cements.

Intraoral self-adhesive resin cements showed different behaviors when used directly on prosthetic materials without their specific primers or associated universal adhesives. GC-O had a completely different behavior between bonding on zirconia or titanium alloy. On zirconia, GC-O with or without its universal silane GMP exhibited almost the same SBS value, whereas significantly low values were observed on Ti6-Al-4V. This could be linked to the absence of MDTP in its composition, whereas this molecule is present on GMP.

For the other self-adhesive resin cements tested without their associated universal primer or universal adhesive on zirconia, we note that there were a number of factors that may explain the lower performance compared to GC-O. Kerr, who produces the OBU and the N-U, is the only brand that uses GPDM instead of 10-MDP in self-adhesive resin cements or dental adhesives. Prior research has shown that 10-MDP has a higher affinity for zirconia than GPDM [44]. Moreover, no synergy seems possible between GPDM and 10-MDP to achieve higher bond strength [48]. Regarding PSA-U and its universal adhesive CUB, the use of the adhesive did not influence the SBS. Bonding on zirconia has lower SBS values despite the presence of MDP in the resin cement and in the adhesive. One explanation could be the higher conversion rate observed for GC-O compared to PSA-U in self-curing mode. Prior research has shown many times that the higher the value, the greater the bond strength values developed for the same product, thanks to improved mechanical properties [49, 50]. TTC has the lowest SBS values on zirconia and Ti-6Al-4V. This result can be explained by the fact that there is no 10-MDP or GPDM in its formula. Rather, its composition includes 4-META, a functional monomer with a high affinity for metal [3], but a lower affinity for zirconia [51], which may explain its better adhesion performance on Ti-6Al-4V than on zirconia. Second, TTC had the lowest conversion rate in dual-cured or self-cured mode of all the other resin cements tested.

### Degree of conversion

All the material in dual cure mode yielded a higher DC compared to the self-cured mode. This outcome has been reported in many studies [52, 53]. Light not only activates the photoinitiators present in the resin but also allows the heat generated by light irradiation to improve the mobility of the resin molecules, thereby increasing the cross-linking rate of the polymerized resin [54].

GPDM is a monomer found only in N-U, which has two methacrylate groups and one phosphate group. These two methacrylate groups can promote better cross-linking in polymerized networks despite other functional monomers, such as 10-MDP [48]. This could explain the observation of this material showing the higher overall DC of N-U in dual-cure.

Dual curing is therefore concluded to optimize the degree of conversion in any clinical or laboratory situation, which can have a major impact on the long-term adhesion stability, particularly in terms of water absorption and resin solubility in the bonding interface [55], which can become infiltrated if light-cured to a limited extent [56, 57]. A high DC results in better mechanical properties and biocompatibility [17, 49, 50, 58].

Surprisingly, MHA, the only extraoral and self-cured resin cement, had higher DC with light curing than self-cure mode. This resin cement is recommended for Ti-base abutment sealing in laboratory and no light-curing is advised. This recommendation is explained by the astonishing presence of camphorquinone, a light curing initiatior in its composition. Therefore, although not recommended by the manufacturer, additional light-curing of this material in the laboratory could result in beneficial properties, especially as the high temperatures caused by possible heating do not appear to alter its mechanical and adhesive properties. This procedure would also be a useful technique for improving its biological properties in the oral environment [19].

This study being in vitro, it includes a certain number of limitations due to this fact, but also to its construction scheme:

- It would be interesting to carry out adhesion tests with ageing, in particular to evaluate the evolution over time of the simplified intra-oral adhesives studied in this study, which contain a certain hydrophilicity due to their self-adhesive chemistry.
- Because of their in vitro nature, these results cannot be directly transposed to the clinic. Long-term studies are needed to draw clinical conclusions.

Finally, as a follow-up to this study, the behavior of the simplified intra-oral adhesives from this study on dental tissues will be investigated in addition to the current investigation on prosthetic tissues.

## Conclusions

In this study, the extraoral self-curing resin cement showed the higher bond strength values on zirconia and Ti-6Al-4V alloy when associated with a universal primer. Some intraoral dual-cure resin cements showed closed performances when used with universal primers.

The extreme simplification of bonding procedures based on the use of self-adhesive resin cements containing functional monomers alone or monomers associated with a universal adhesive led to variable results depending on brands and formulations. These results may nevertheless be sufficient for these applications given that they yielded bond strength values close to those of conventional procedures.

There is no direct correlation between the degree of conversion of the resin cement and the shear bond strength obtained on the prosthetic materials tested. Additional light curing is advised in every case of bonding to enhance the degree of conversion.

However, further clinical studies validating these results are required before generalizing these conclusions.

## Data Availability

The data that support the findings of this study are available from the corresponding author upon reasonable request.
